# Impact of Alcohol on Inflammation, Immunity, Infections, and Extracellular Vesicles in Pathogenesis

**DOI:** 10.7759/cureus.56923

**Published:** 2024-03-25

**Authors:** Jayaraman Tharmalingam, Prakash Gangadaran, Ramya Lakshmi Rajendran, Byeong-Cheol Ahn

**Affiliations:** 1 Department of Biomedical Engineering, University of Houston, Houston, USA; 2 Department of Nuclear Medicine, Kyungpook National University, Daegu, KOR; 3 Department of Biomedical Science, BK (Brain Korea) 21 FOUR (Fostering Outstanding Universities for Research) Program, Kyungpook National University, Daegu, KOR; 4 Department of Nuclear Medicine, Kyungpook National University Hospital, Daegu, KOR

**Keywords:** extracellular vesicles, liver, infections, immunity, inflammation, alcohol

## Abstract

Alcohol consumption is a widespread social activity with a complex and multifaceted impact on human health. Although moderate alcohol consumption has been associated with certain potential health benefits, excessive or chronic alcohol use can disrupt the body’s immune balance, promote inflammation, and increase susceptibility to infections. The deleterious effects associated with alcohol toxicity include the loss of cell integrity. When cells lose their integrity, they also lose the capacity to communicate with other systems. One of the systems disturbed by alcohol toxicity is extracellular vesicle (EV)-mediated communication. EVs are critical mediators of cell-to-cell communication. They play a significant role in alcohol-induced pathogenesis, facilitating communication and molecular exchange between cells, thereby potentially contributing to alcohol-related health issues. Investigating their involvement in this context is fundamental to resolving the intricate mechanisms behind the health consequences of alcohol use and may pave the way for innovative approaches for mitigating the adverse effects of alcohol on immune health. Understanding the role of EVs in the context of alcohol-induced pathogenesis is essential for comprehending the mechanisms behind alcohol-related health issues.

## Introduction and background

Alcohol and inflammation

Alcohol is one of the most abused substances worldwide irrespective of the socioeconomic status of the countries. Almost half of substance abusers are alcoholic and require medical attention for treating alcohol-related organ damage and infections. Annually, alcohol abuse accounts for approximately 3.3 million deaths worldwide and it is the fifth most common cause of death in the United States and Europe [1].

Alcohol consumption varies across gender and race/ethnicity. Worldwide, men consume more alcohol than women, and American men are much more likely than women to use alcohol, binge drink, and report heavy drinking. Among racial and ethnic groups in the United States, White individuals report the highest overall alcohol use, with alarming trends in alcohol misuse observed among both genders and various ethnicities over the past decade [2].

Alcohol abuse can cause injuries to several vital organs, including the liver, brain, gut, pancreas, and lungs. Tissue injuries are caused by oxidative stress, inflammation, and impaired immune responses due to alcohol metabolites [3]. The importance of this review lies in its contribution to understanding the multifaceted impacts of alcohol on inflammation, immunity, infections, and extracellular vesicles (EVs) in pathogenesis. The primary purpose of conducting this review is to consolidate and analyze existing literature to elucidate the complex mechanisms underlying alcohol's effects on these key physiological processes. This review aims to provide an overview of the interplay between alcohol consumption and its consequences on inflammatory responses, immune function, susceptibility to infections, and EV-related dynamics.

## Review

Alcohol enhances inflammation

Alcohol causes injuries to various tissues, which can reach up to cellular levels. Alcoholic metabolite-induced breakdown of cell walls generates reactive oxygen species (ROS) formation. ROS can activate nuclear factor-κB (NF-κB), a key inflammation-associated transcription factor [4]. Cell injury action of inflammation is mostly driven by innate immune cells, including macrophages, antigen-presenting cells, and neutrophils. Innate immune response, a nonspecific response, attacks foreign invaders or injurious sites to increase blood flow and subsequently facilitate cellular repair. Uncontrolled inflammation is deleterious to tissues and various vital organs.

Alcohol-induced uncontrolled inflammation is one of the main reasons for several chronic inflammatory diseases. Alcoholic liver disease (ALD) is caused by inflammation in the liver, particularly owing to the increased pro-inflammatory cytokine response in the liver [5]. Alcohol-induced pro-inflammatory cytokine, TNF-α, is the most significant factor for liver disease. The role of alcohol-induced cytokine TNF-α-associated inflammation in liver cirrhosis was established in a TNF-α gene knockdown mouse, which was resistant to alcoholic liver fibrosis/cirrhosis [6].

The reason for the poor recovery associated with ALD is the increase in serum inflammatory cytokine TNF-α level, which is caused by circulatory monocytes. Alcohol consumption alters the levels of macrophage colony-stimulating factor (essential for monocyte development and differentiation) in the serum, which increases the inflammatory monocyte than monocyte-derived macrophage and is one of the reasons for ALD [7].

Other alcohol consumption-related morbidities were well documented in human history. Dr. Robert Koch, a well-known microbiologist and historian, observed that alcoholic individuals had more bacterial infection-associated morbidities than their healthy counterparts [8]. Several reviews have reported that alcoholic participants are prone to bacterial and viral infections [9,10]. Alcohol-altered or -suppressed immune response in alcoholic individuals is one of the main reasons for their susceptibility to infections. It was proven in an animal alcoholic model that acute administration of alcohol increases corticosterone levels in the blood; corticosterone is a steroid that is associated with the reduction in lymphocyte levels in mice [11].

Inflammation is a continuing process in several injurious conditions, including liver cirrhosis or gut injuries, due to the constant influx of pro-inflammatory cytokines produced by infiltrated macrophages and neutrophils [12]. Alcohol-induced ROS production leads to the activation of inflammation gene-specific NF-κB transcription factor and inflammasome signaling pathways [13,14]. IL-18, a cytokine specific to inflammasomes, and caspase-1, one of the inflammasome components, were increased in the alcohol-treated rats, which led to increased inflammation in the injured brain tissue [15]. Chronic alcohol consumption leads to cellular injuries, and constant inflammation leads the normal cells to turn cancerous. Alcohol can influence cellular signaling that turns normal cells into tumor cells in an animal model [16].

Chronic alcohol consumption alters the composition and growth of the gut microbiota which helps the gram-negative bacterial growth and increases the circulatory lipopolysaccharide (LPS) [17,18]. Human and animal studies have shown that with or without liver diseases, acute alcohol consumption significantly increases circulatory LPS levels; endotoxin replicates the impact of ethanol on oxygen consumption, and endotoxin plays a pivotal role in the rapid increase in alcohol metabolism by stimulating eicosanoid release from Kupffer cells (KCs) [19,20]. Nitro-oxidative stress due to inducible nitric oxide synthase (iNOS) expression, Nf-κB signaling activation, and microRNA-122 (miRNA-122) expression in the intestinal cells due to alcohol are the main reasons for altered gut permeability [21,22]. Increased LPS accumulation in the liver leads to the activation of KCs [23]. Leaked LPS acts on liver tissues and immune cells in the liver, particularly the KCs, through the toll-like receptor (TLR) signaling pathway to increase the levels of inflammatory cytokines, including TNF-α and IL-1β, which are the sources of inflammation-induced ALD [5,24,25]. Adachi et al. reported that the deletion of KCs in the liver prevents the development of liver disease in an alcohol-induced liver disease model [26].

Alcohol enhances immune imbalance and infection

The human immune system responds to invading pathogens in the following two ways: (1) nonspecific innate and (2) specific adaptive immune response. The innate immune system recognizes pathogen-associated molecular patterns (PAMPs) and produces inflammatory mediators, specifically pro-inflammatory mediators, to control infections. Most of the pro-inflammatory mediators are considered double-edged swords as they are well known for their protective response against infection; however, they were reportedly causative in several diseases, including liver cirrhosis, chronic obstructive pulmonary disease, and other gut-associated inflammatory diseases due to tissue damage by uncontrolled inflammation [27]. The immune system and other tissues produce anti-inflammatory mediators that check and counter pro-inflammatory mediators and the deleterious effects of inflammation. To avoid cellular injuries, a balance between pro-inflammatory and anti-inflammatory responses is significant. Several external agents are acute and chronically alter this balance, and one of such agents is alcohol.

Alcohol not only alters the balance of inflammatory mediators but also alters the responders (macrophages, neutrophils, eosinophils, mast cells, T cells, and B cells) of the immune system to make the host susceptible to infections [28,29].

**Figure 1:**
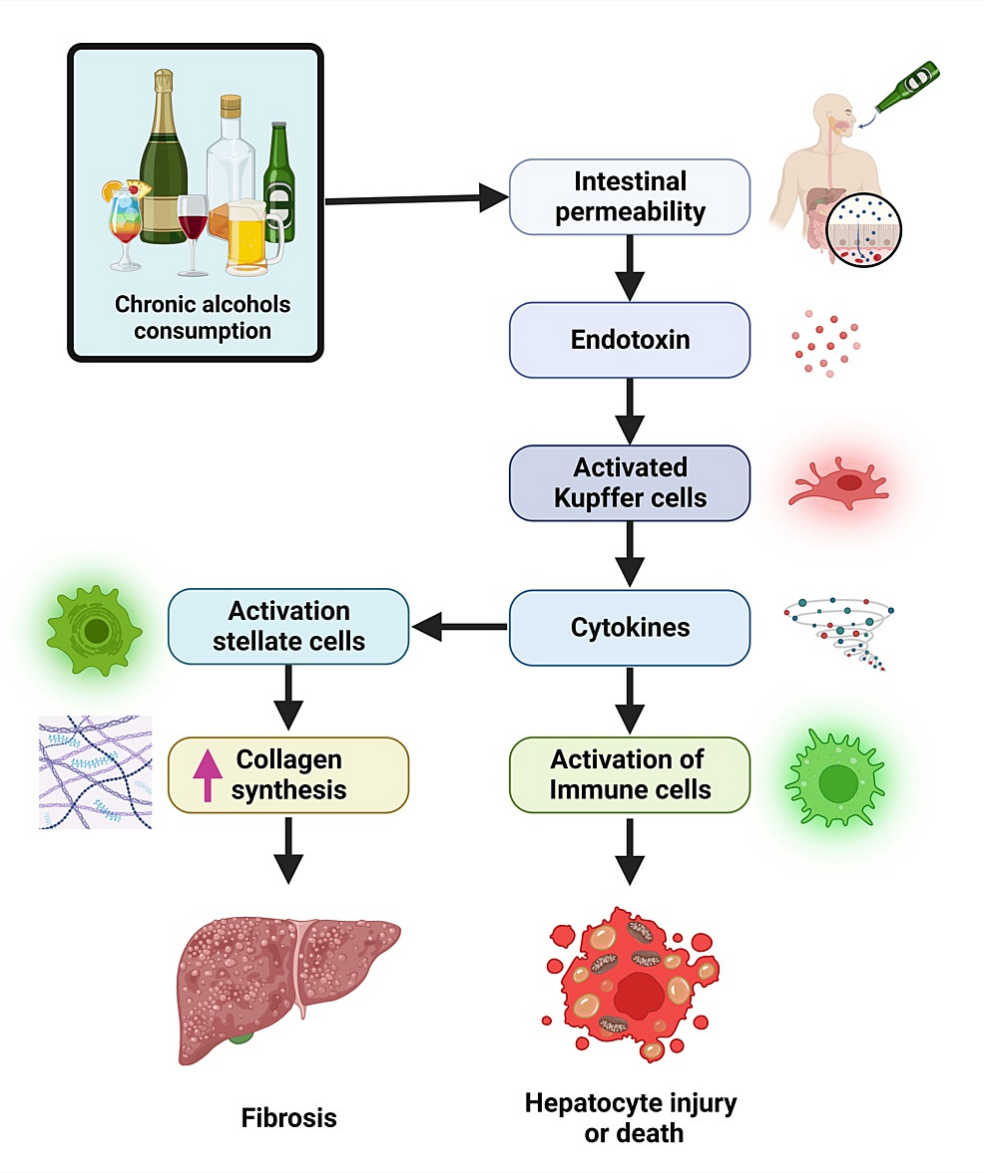
Pathological processes involved in alcoholic liver disease. Chronic alcohol consumption increases gut permeability, allowing endotoxins to enter the liver and trigger inflammation. This inflammation leads to fibrosis. Cytokines released from the Kupffer cells further activate immune cells and lead to hepatocyte injury or death. Image created using BioRender.com

Alcohol-induced imbalance in pro-inflammatory and anti-inflammatory responses is associated with inflammatory diseases or severe infection under suppressed immune response against infection. Reduced levels of the anti-inflammatory cytokine IL-10 lead to the production of pro-inflammatory cytokine TNF-α, which increases liver injury in chronic alcoholics [30]. Reduced IL-10 levels in the circulation increase IL-6 levels, which activates pro-inflammatory Th17 response in the liver, thereby leading to inflammatory liver disease (Figure 1) [31].

Acute alcohol exposure decreases the protective pro-inflammatory response against gram-negative bacteria LPS by reduced transactivation of p50 complex in the Nf-κB transcription factor. The NF-κB signaling pathway is important for inflammatory signaling activation in several innate immune cells [32]. TNF-α prevents several bacterial infections; however, the action of TNF-α is impaired during acute exposure to alcohol [33]. Alcohol directly alters TNF-α function by inhibiting pro-TNF-α conversion into the active form [34]. Bacterial antigen LPS impairs the action of reduced inflammatory cytokine TNF-α in the lungs, thereby leading to reduced neutrophil infiltration that increases bacterial load in acute alcohol-fed mice lungs [33].

Neutrophil, one of the innate immune system components, is essential for the clearance of infection. Alcohol exposure impairs the neutrophil activity against infections. The granulocyte colony-stimulating factor (G-CSF) is crucial for the activation of several granulocytes, including neutrophils. In alcoholic conditions, the expression patterns of G-CSF are altered, thereby leading to reduced neutrophil accumulation, potentially leading to pneumonia infection [35,36]. Inflammation caused by infiltrated neutrophils and monocytes is the major reason for alcohol-induced liver injury [37,38].

Alcoholic individuals are susceptible to infection owing to altered immune response. Pneumonia is one of the bacterial infections caused by the alcohol-altered immune response. Schmidt et al. observed that alcoholic individuals are more susceptible and have higher mortality due to pneumonia than non-alcoholic individuals [39]. Community-acquired pneumonia and septic shock in alcohol-abused individuals are caused by altered immune responses against *Pseudomonas aeruginosa* and *Acinetobacter* species [40]. *P. aeruginosa* is an opportunistic infection in alcoholic individuals that is caused by altered inflammatory nitric oxide production in the neutrophil, thereby leading to severe respiratory infection [41]. The inability of innate immune cells to recognize several pathogenic and non-pathogenic bacteria is because of the alcohol-altered innate immune cell TLRs' recognition of PAMP and suppression of several inflammatory cytokines [42].

Several epidemiological studies showed that alcohol abuse is the most significant reason for several pulmonary infections, particularly of *Mycobacterium tuberculosis*, owing to alcohol-altered host immune response against infection [43]. Chronic alcohol use alters the bacterial clearance capacity of alveolar macrophages by reducing phagocytic activity, which along with superoxide production, is significant for infection clearance [41,44]. Sachs et al. showed that reduced superoxide production and the altered phagocytic activity of neutrophils increase the host’s susceptibility to bacterial infections [45].

Acute alcohol impairs chemokine expression in the lungs, particularly the neutrophil-attracting chemokine CXCL 1 KC and growth-related oncogene-alpha (GRO-α). Reduced neutrophil infiltration leads to bacterial infection following viral pneumonia in chronic alcohol users [46,47]. Chronic alcohol feeding in an animal model showed that alveolar macrophages impaired the clearance of Staphylococcus aureus infection owing to reduced cellular glutathione, increased lipid peroxidation, and alveolar macrophage apoptosis [48]. IL-23, a significant mediator for Th17 immune response development, is suppressed in lung and alveolar macrophages following acute alcoholic treatment. The lung innate immune system cannot handle bacterial infection clearance without IL-23 [49]. Alcohol and alcohol-associated action about alcohol-mediated causes are tabulated in Table 1. 

**Table 1:** Alcoholic condition with associated action and effects. TNF-α: tumor necrosis factor-alpha; IL: interleukin; MCP1: monocyte chemoattractant protein-1; Hmgb1: high mobility group box 1; NF-κB: nuclear factor kappa B; GRO-α: growth-related oncogene-alpha; HCV: hepatitis C virus; LPS: lipopolysaccharide; ALD: alcoholic liver disease; DC: dendritic cell

Alcoholic condition	Alcohol-associated action	Alcohol-associated effect
Alcoholism [24]	Activated Kupffer cells-associated TNF-a	Alcoholic fatty disease
ALD [7]	Increases macrophage colony-stimulating factor	Increased inflammatory monocytes lead to ALD
Chronic alcoholism [9]	Alcohol-associated reduction in granulocyte colony-stimulating factor and IL-12	*Klebsiella pneumonia *infection
Chronic alcoholism [9]	Alcohol-induced hepatocyte necrosis, infiltration of inflammatory cells	Severity of bacterial infection* Listeria* and *Borrellia *increases
Alcoholism and HCV infection [10]	Alcohol-induced oxidative stress alters the mitochondrial membrane and reduces antioxidants, increases viral infection	HCV infection
Alcoholism [10]	Reduces the DC-associated reduction of IL-12 and increases IL-10	Impaired DC leads to severe HCV infection
Alcoholism [11]	Increases the corticosterone levels and reduces lymphocytes	Immunosuppression
Alcoholism and injuries [15]	Alcohol increases the inflammasome-associated IL-18 and neutrophil in injuries	Severity of injuries increases
Chronic alcoholism [21]	Alcohol increases nitric oxide, NF-κB signalling and intestinal-associated microRNA-122	Permeability of gut and circulating LPS-associated inflammation increases
Alcoholic cirrhosis [31]	Alcohol-induced IL-17, IL-8 and GRO-α-associated neutrophil recruitment and injuries	ALD
Alcohol-associated infection [34]	Alcohol inhibits the conversion of pro-TNF-a to active form	Bacterial infection
Alcohol-associated infection [43,48]	Alcohol-altered alveolar macrophage phagocytic activity and superoxide dismutase	Bacterial infection (*Mycobacterium* infection)
Chronic alcoholism [49]	Alcohol reduces IL-23 and associated Th17 response	Bacterial infection

Alcoholic conditions not only influence innate immune response but also influence adaptive immune response by reduced lymphocyte content in the thymus due to reduced thymic size in chronic alcoholic individuals [50]. Ethanol-fed animals are seen to have impaired T cell response against mitogen antigens and delayed-type hypersensitivity response [51,52]. CD4+ T cell counts in the mucosal immune system are altered in an experimental alcoholic intoxication non-human primate model [53].

Alcohol potentially alters the monocytes and monocyte-derived dendritic cells' (DCs') inability to activate T cell response is a major factor in alcohol-related infectious diseases. Altered DCs produce more anti-inflammatory IL-10, and reduced IL-12 levels lead to the development of various infections [54]. The expression of CD80/86, which are surface markers of DCs, are stunted, and the levels of bone marrow-derived DCs are decreased in alcoholic-fed mice, which are unable to mount T cell activation and drive inflammatory IL-12 cytokine [55].

T cell response is essential for viral infection clearance. Alcohol has the potential to modulate T cell response activation. A moderate dose of alcohol along with viral infection, particularly hepatitis C virus (HCV), can modulate or reduce DCs’ interaction with T cell for T cell activation. This reduced DC activation is because of IL-12 production from other cells under alcoholic intoxication. This is another reason for alcoholic individuals’ increased susceptibility to developing severe liver infection by HCV [54]. Anti-viral cytokine IFN-γ production in the innate and adaptive immune systems is severely impaired in alcohol-fed mice [56]. HCV infection is one of the major causes of liver cirrhosis along with alcohol. Alcohol along with HCV increases the susceptibility to liver diseases in the following three ways: (1) alcohol-stimulated viral replication, (2) alcohol-associated dysfunctional immune response, and (3) alcohol-induced oxidative stress in the mitochondria [57].

HIV infection progression depends on CD4 T cell counts, and anti-retroviral treatment reportedly depends on the use or withdrawal of alcohol consumption [58]. An animal model of simian immunodeﬁciency virus infection showed that alcohol impairs the nutritional status and increases TNF-α in muscles to cause muscle wasting [59].

Alcohol-fed animals showed that reduced T cell proliferation and altered CD4 and CD8 T cell counts were major reasons for pulmonary tuberculosis in infected animals [60]. Animal studies reported that alcohol intoxication leads to suppressed pro-inflammatory cytokines, such as IL-12 and interferon‐gamma (IFN‐γ); however, this pro-inflammatory suppression due to alcohol mediated the increase in anti-inflammatory cytokine IL-10 [61,62]. Alcohol intoxication, which inhibits the IL-17 cytokine production in T cells, helps establish bacterial infection; however, the addition of external IL-17 reverses the immune cell functions and clears the bacterial infection [63,64]. In patients with ALD, there is reduced IgG and IgG1 B cell levels in the blood and impaired T cell-dependent B cell response as opposed to T cell-independent B cell response [65,66]. Alcoholic-abusive individuals are more susceptible to influenza infection owing to an alternated inflammatory environment in the lungs along with decreased CD8 T cell counts as observed in chronic alcohol-fed mice [67].

EVs in alcohol-induced pathogenesis

EVs are small vesicles secreted by cells, and they play a role in intercellular communication by transporting various molecules, including proteins, nucleic acids, and lipids [68,69]. Although studies on the specific contributions of EVs to alcohol’s effects on inflammation, immune imbalance, and infection are ongoing, some evidence suggests that EVs play a role in these processes. Alcoholic hepatitis (AH) is primarily driven by hepatocyte damage and inflammation, wherein miRNA-122 plays a pivotal role. Although hepatocytes have an abundance of miRNA-122 [70-72], the interplay between hepatocyte-derived exosomes and immune cells in AH remains unexplored. A study reported a substantial increase in exosome levels in both healthy individuals following binge drinking and mice consuming alcohol. Ethanol-treated hepatocytes in vitro realized increased levels of exosomes, which contain miRNA-122. Treatment of THP1 monocytes with hepatocyte-derived exosomes containing miRNA-122 resulted in the delivery of mature miRNA-122, leading to the inhibition of the HO-1 pathway. Sensitization of THP1 monocytes with LPS stimulation resulted in increased levels of pro-inflammatory cytokines. RNA interference, facilitated by exosome delivery, mitigates the inflammatory effects of exosomes from ethanol-treated hepatocytes, underscoring the role of exosomes in mediating communication between hepatocytes and monocytes and the miRNA-122-induced monocyte reprogramming [73].

Alcohol exposure leads to a concentration- and time-dependent increase in EV production, primarily exosomes, by human monocytes and THP-1 monocytic cells. These alcohol-induced EVs trigger naive monocytes to transform into M2 macrophages, as indicated by the increased expression of macrophage markers (CD68, CD206, and CD163), IL-10, and transforming growth factor-β (TGF-β) secretion, and enhance phagocytic activity. Furthermore, profiling the miRNA in alcohol-exposed THP-1 monocyte-derived EVs shows increased levels of miR-27a, an M2-polarizing miRNA. In a study by Saha et al., treating naive monocytes with miR-27a-overexpressing control EVs replicated the impact of EVs from alcohol-exposed monocytes, inducing M2 polarization, indicating that miR-27a mediated the effects of alcohol EVs [74]. In a study by Saha et al., a notable elevation in the total count of circulating EVs was noted in mice ALD compared with the pair-fed control group [75]. Mass spectrometric analysis of these circulating EVs unveiled a unique protein signature, indicating involvement in inflammatory responses, cellular development, and cellular movement, distinguishing ALD EVs from control EVs. ALD EV-recipient mice exhibited elevated quantities of F4/80hi CD11blo KCs and higher proportions of inflammatory/M1 KCs expressing TNF-α and IL-12/23, as well as infiltrating monocytes (F4/80intCD11bhi). Conversely, the percentage of anti-inflammatory/M2 KCs marked by CD206 and CD163 was reduced compared with that of the control EV-recipient mice. Furthermore, they also identified heat shock protein 90 present in ALD EVs as the mediator responsible for activating macrophages in response to ALD EVs [75].

**Figure 2:**
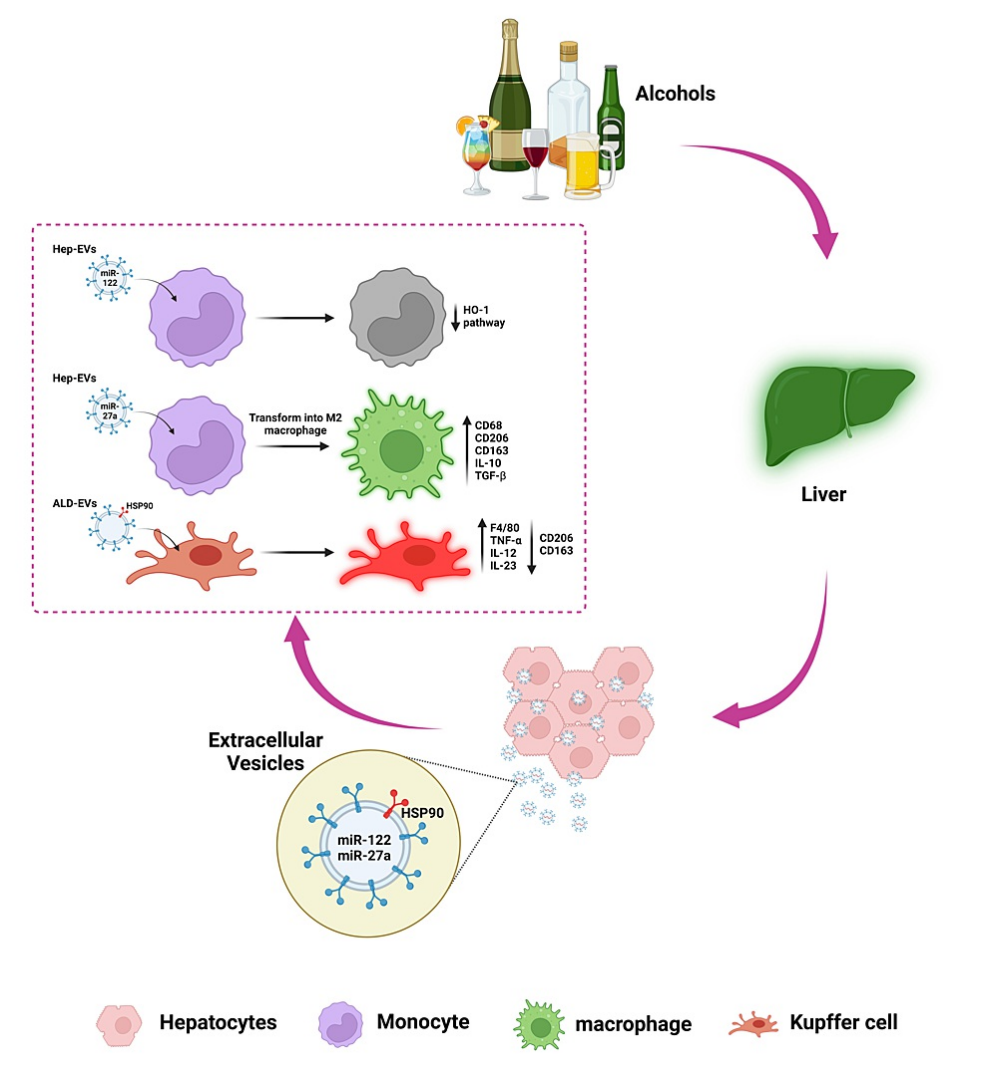
Role of extracellular vesicles in alcohol-induced pathogenesis EVs are portrayed as small vesicular structures traversing the extracellular space/bodily fluids. EVs act as messengers, facilitating communication between hepatocytes and immune cells. Alcohol-induced/consumed hepatocyte-derived EVs (Hep-EVs)/ALD mice-derived EVs (ALD EVs) contain cargoes (miR122, miR27a, and HSP90 protein) traversing into monocyte/Kupffer cells, thereby leading to cellular alterations (e.g., decreased HO-1 pathway in monocytes; monocyte transforms into M2 macrophages by elevating CD68, CD206, CD163, IL-10, and TGF-β levels; and elevation of F4/80, TNF-α, IL-12 and IL-23/decrease of CD206 and CD163 in Kupffer cells) and inflammation. EV: extracellular vesicle; CD: cluster of differentiation; IL: interleukin; TGF-β: transforming growth factor-beta; TNF-α: tumor necrosis factor-alpha; ALD: alcoholic liver disease Image created using BioRender.com

In a study by Momen-Heravi et al., a significant increase was observed in the number of circulating EVs following alcohol consumption in mice, which were primarily composed of exosomes, a smaller subcategory of EVs [76]. Analyzing these exosomes using microarray screening, they identified nine inflammatory miRNAs with altered expression in mice with chronic alcohol consumption compared with the control mice. Notably, miRNA-192, miRNA-122, and miRNA-30a were upregulated. ROC analysis confirmed that miRNA-192, miRNA-122, and miRNA-30a had strong diagnostic potential for detecting alcohol-induced liver injury. Subsequently, these findings were validated in human samples, where a similar increase in total EVs, mainly exosomes, was observed in individuals with AH. Furthermore, both miRNA-192 and miRNA-30a showed significant elevation in patients with AH, with miRNA-192 holding promise as a diagnostic marker for AH [76]. According to the available literature, alterations in cellular protein and mRNA due to alcohol align with corresponding changes in cargoes carried by EVs (Figure 2). This biomolecule exchange between cells leads to heightened or reduced inflammatory responses in the receiving cells. Gaining a deeper comprehension of the interaction between EVs and alcohol holds the potential for enhanced personalized healthcare for individuals who partake in its consumption.

## Conclusions

Alcohol abuse has direct effects on the cell membrane that damage several cellular protective mechanisms, thereby leading to oxidative stress, which is one of the significant causes of various diseases caused by alcohol abuse. The increase in severity and the occurrence of other comorbidities due to alcohol abuse are caused by the indirect effects of alcohol on the cellular inflammatory mechanism and immune system. It is important for the clinician to take note of the patient’s pre-history of alcohol use as clinical parameters vary due to alcohol-induced inflammation. Armed with this knowledge, physicians can better counsel patients on the risks associated with excessive alcohol intake and tailor preventative measures to mitigate inflammation-related complications and infectious diseases. Moreover, the elucidation of alcohol's influence on EVs offers novel avenues for therapeutic intervention and underscores the importance of holistic approaches in managing alcohol-related pathologies. Through multiple studies, it has been elucidated that alcohol intake disrupts the body's immune response, leading to chronic inflammation and impaired defense mechanisms against pathogens. EVs emerge as crucial participants in the intricate web of alcohol-induced pathologies, and understanding their role may pave the way for more targeted strategies for mitigating the impact of alcohol on inflammation, immune imbalance, and infection.

## References

[1] Ohashi K, Pimienta M, Seki E (2018). Alcoholic liver disease: a current molecular and clinical perspective. Liver Res.

[2] Sudhinaraset M, Wigglesworth C, Takeuchi DT (2016). Social and cultural contexts of alcohol use: influences in a social-ecological framework. Alcohol Res.

[3] Rusyn I, Bataller R (2013). Alcohol and toxicity. J Hepatol.

[4] Nowak AJ, Relja B (2020). The impact of acute or chronic alcohol intake on the NF-κB signaling pathway in alcohol-related liver disease. Int J Mol Sci.

[5] McClain CJ, Barve S, Deaciuc I, Kugelmas M, Hill D (1999). Cytokines in alcoholic liver disease. Semin Liver Dis.

[6] Yin M, Wheeler MD, Kono H, Bradford BU, Gallucci RM, Luster MI, Thurman RG (1999). Essential role of tumor necrosis factor α in alcohol-induced liver injury in mice. Gastroenterology.

[7] Alharshawi K, Fey H, Vogle A, Klenk T, Kim M, Aloman C (2021). Alcohol consumption accumulation of monocyte derived macrophages in female mice liver is interferon alpha receptor dependent. Front Immunol.

[8] Howard-Jones N (1984). Robert Koch and the cholera vibrio: a centenary. Br Med J (Clin Res Ed).

[9] Pavia CS, La Mothe M, Kavanagh M (2004). Influence of alcohol on antimicrobial immunity. Biomed Pharmacother.

[10] Szabo G, Aloman C, Polyak SJ, Weinman SA, Wands J, Zakhari S (2006). Hepatitis C infection and alcohol use: a dangerous mix for the liver and antiviral immunity. Alcohol Clin Exp Res.

[11] Collier SD, Wu WJ, Pruett SB (1998). Endogenous glucocorticoids induced by a chemical stressor (ethanol) cause apoptosis in the spleen in B6C3F1 female mice. Toxicol Appl Pharmacol.

[12] Brunt PW, Kew MC, Scheuer PJ, Sherlock S (1974). Studies in alcoholic liver disease in Britain. I. Clinical and pathological patterns related to natural history. Gut.

[13] Gloire G, Legrand-Poels S, Piette J (2006). NF-kappaB activation by reactive oxygen species: fifteen years later. Biochem Pharmacol.

[14] Lowe PP, Gyongyosi B, Satishchandran A, Iracheta-Vellve A, Cho Y, Ambade A, Szabo G (2018). Reduced gut microbiome protects from alcohol-induced neuroinflammation and alters intestinal and brain inflammasome expression. J Neuroinflammation.

[15] Li X, Kovacs EJ, Schwacha MG, Chaudry IH, Choudhry MA (2007). Acute alcohol intoxication increases interleukin-18-mediated neutrophil infiltration and lung inflammation following burn injury in rats. Am J Physiol Lung Cell Mol Physiol.

[16] Machida K, Tsukamoto H, Mkrtchyan H (2009). Toll-like receptor 4 mediates synergism between alcohol and HCV in hepatic oncogenesis involving stem cell marker Nanog. Proc Natl Acad Sci U S A.

[17] Bode JC, Bode C, Heidelbach R, Dürr HK, Martini GA (1984). Jejunal microflora in patients with chronic alcohol abuse. Hepatogastroenterology.

[18] Hauge T, Persson J, Danielsson D (1997). Mucosal bacterial growth in the upper gastrointestinal tract in alcoholics (heavy drinkers). Digestion.

[19] Bode C, Kugler V, Bode JC (1987). Endotoxemia in patients with alcoholic and non-alcoholic cirrhosis and in subjects with no evidence of chronic liver disease following acute alcohol excess. J Hepatol.

[20] Rivera CA, Bradford BU, Seabra V, Thurman RG (1998). Role of endotoxin in the hypermetabolic state after acute ethanol exposure. Am J Physiol.

[21] Ferrier L, Bérard F, Debrauwer L, Chabo C, Langella P, Buéno L, Fioramonti J (2006). Impairment of the intestinal barrier by ethanol involves enteric microflora and mast cell activation in rodents. Am J Pathol.

[22] Tang Y, Banan A, Forsyth CB, Fields JZ, Lau CK, Zhang LJ, Keshavarzian A (2008). Effect of alcohol on miR-212 expression in intestinal epithelial cells and its potential role in alcoholic liver disease. Alcohol Clin Exp Res.

[23] Enomoto N, Ikejima K, Yamashina S (2001). Kupffer cell sensitization by alcohol involves increased permeability to gut‐derived endotoxin. Alcohol Clin Exp Res.

[24] Gustot T, Lemmers A, Moreno C (2006). Differential liver sensitization to toll-like receptor pathways in mice with alcoholic fatty liver. Hepatology.

[25] Duryee MJ, Klassen LW, Freeman TL, Willis MS, Tuma DJ, Thiele GM (2004). Lipopolysaccharide is a cofactor for malondialdehyde-acetaldehyde adduct-mediated cytokine/chemokine release by rat sinusoidal liver endothelial and Kupffer cells. Alcohol Clin Exp Res.

[26] Adachi Y, Bradford BU, Gao W, Bojes HK, Thurman RG (1994). Inactivation of Kupffer cells prevents early alcohol‐induced liver injury. Hepatology.

[27] Furman D, Campisi J, Verdin E (2019). Chronic inflammation in the etiology of disease across the life span. Nat Med.

[28] Brown LA, Cook RT, Jerrells TR (2006). Acute and chronic alcohol abuse modulate immunity. Alcohol Clin Exp Res.

[29] Szabo G (1999). Consequences of alcohol consumption on host defence. Alcohol Alcohol.

[30] McClain CJ, Cohen DA (1989). Increased tumor necrosis factor production by monocytes in alcoholic hepatitis. Hepatology.

[31] Lemmers A, Moreno C, Gustot T (2009). The interleukin-17 pathway is involved in human alcoholic liver disease. Hepatology.

[32] Mandrekar P, Jeliazkova V, Catalano D, Szabo G (2007). Acute alcohol exposure exerts anti-inflammatory effects by inhibiting IkappaB kinase activity and p65 phosphorylation in human monocytes. J Immunol.

[33] Nelson S, Bagby G, Summer WR (1989). Alcohol suppresses lipopolysaccharide-induced tumor necrosis factor activity in serum and lung. Life Sci.

[34] Zhao XJ, Marrero L, Song K (2003). Acute alcohol inhibits TNF-alpha processing in human monocytes by inhibiting TNF/TNF-alpha-converting enzyme interactions in the cell membrane. J Immunol.

[35] Bagby GJ, Zhang P, Stoltz DA, Nelson S (1998). Suppression of the granulocyte colony‐stimulating factor response to escherichia coli challenge by alcohol intoxication. Alcohol Clin Exp Res.

[36] Nelson S, Belknap SM, Carlson RW (1998). A randomized controlled trial of filgrastim as an adjunct to antibiotics for treatment of hospitalized patients with community-acquired pneumonia. CAP Study Group. J Infect Dis.

[37] Xu R, Huang H, Zhang Z, Wang FS (2014). The role of neutrophils in the development of liver diseases. Cell Mol Immunol.

[38] Wang M, You Q, Lor K, Chen F, Gao B, Ju C (2014). Chronic alcohol ingestion modulates hepatic macrophage populations and functions in mice. J Leukoc Biol.

[39] Schmidt W, Lint J de (1972). Causes of death of alcoholics. Q J Stud Alcohol.

[40] Marik PE (2000). The clinical features of severe community-acquired pneumonia presenting as septic shock. Norasept II Study Investigators. J Crit Care.

[41] Greenberg SS, Zhao X, Hua L, Wang J-F, Nelson S, Ouyang J: Ethanol Inhibits Lung (1999). Clearance of Pseudomonas aeruginosa by a neutrophil and nitric oxide-dependent mechanism, in vivo. Alcohol Clin Exp Res.

[42] Pruett SB, Zheng Q, Fan R, Matthews K, Schwab C (2004). Ethanol suppresses cytokine responses induced through Toll-like receptors as well as innate resistance to Escherichia coli in a mouse model for binge drinking. Alcohol.

[43] Moran A, Harbour DV, Teeter LD, Musser JM, Graviss EA (2007). Is alcohol use associated with cavitary disease in tuberculosis?. Alcohol Clin Exp Res.

[44] Greenberg SS, Ouyang J, Zhao X, Parrish C, Nelson S, Giles TD (1999). Effects of ethanol on neutrophil recruitment and lung host defense in nitric oxide synthase I and nitric oxide synthase ii knockout mice. Alcohol Clin Exp Res.

[45] Sachs CW, Christensen RH, Pratt PC, Lynn WS (1990). Neutrophil elastase activity and superoxide production are diminished in neutrophils of alcoholics. Am Rev Respir Dis.

[46] Zhang P, Bagby GJ, Xie M, Stoltz DA, Summer WR, Nelson S (1998). Acute ethanol intoxication inhibits neutrophil β2‐integrin expression in rats during endotoxemia. Alcohol Clin Exp Res.

[47] Navarini AA, Recher M, Lang KS (2006). Increased susceptibility to bacterial superinfection as a consequence of innate antiviral responses. Proc Natl Acad Sci U S A.

[48] Brown LA, Ping XD, Harris FL, Gauthier TW (2007). Glutathione availability modulates alveolar macrophage function in the chronic ethanol-fed rat. Am J Physiol Lung Cell Mol Physiol.

[49] Happel KI, Odden AR, Zhang P, Shellito JE, Bagby GJ, Nelson S (2006). Acute alcohol intoxication suppresses the interleukin 23 response to Klebsiella pneumoniae infection. Alcohol Clin Exp Res.

[50] Saad AJ, Jerrells TR (1991). Flow cytometric and immunohistochemical evaluation of ethanol-induced changes in splenic and thymic lymphoid cell populations. Alcohol Clin Exp Res.

[51] Spinozzi F, Bertotto A, Rondoni F, Gerli R, Scalise F, Grignani F (1991). T-lymphocyte activation pathways in alcoholic liver disease. Immunology.

[52] Lundy J, Raaf JH, Deakins S (1975). The acute and chronic effects of alcohol on the human immune system. Surg Gynecol Obstet.

[53] Poonia B, Nelson S, Bagby GJ, Veazey RS (2006). Intestinal lymphocyte subsets and turnover are affected by chronic alcohol consumption: implications for SIV/HIV infection. J Acquir Immune Defic Syndr.

[54] Mandrekar P, Catalano D, Dolganiuc A, Kodys K, Szabo G (2004). Inhibition of myeloid dendritic cell accessory cell function and induction of T cell anergy by alcohol correlates with decreased IL-12 production. J Immunol.

[55] Lau AH, Abe M, Thomson AW (2006). Ethanol affects the generation, cosignaling molecule expression, and function of plasmacytoid and myeloid dendritic cell subsets in vitro and in vivo. J Leukoc Biol.

[56] Jerrells TR, Mitchell K, Pavlik J, Jerrells J, Hoerman D (2002). Influence of ethanol consumption on experimental viral hepatitis. Alcohol Clin Exp Res.

[57] Zhang T, Li Y, Lai JP, Douglas SD, Metzger DS, O'Brien CP, Ho WZ (2003). Alcohol potentiates hepatitis C virus replicon expression. Hepatology.

[58] Miguez MJ, Shor-Posner G, Morales G, Rodriguez A, Burbano X (2003). HIV treatment in drug abusers: impact of alcohol use. Addict Biol.

[59] Molina PE, McNurlan M, Rathmacher J (2006). Chronic alcohol accentuates nutritional, metabolic, and immune alterations during asymptomatic simian immunodeficiency virus infection. Alcohol Clin Exp Res.

[60] Mason CM, Dobard E, Zhang P, Nelson S (2004). Alcohol exacerbates murine pulmonary tuberculosis. Infect Immun.

[61] Zisman DA, Strieter RM, Kunkel SL, Tsai WC, Wilkowski JM, Bucknell KA, Standiford TJ (1998). Ethanol feeding impairs innate immunity and alters the expression of Th1- and Th2-phenotype cytokines in murine Klebsiella pneumonia. Alcohol Clin Exp Res.

[62] Szabo G, Mandrekar P, Dolganiuc A, Catalano D, Kodys K (2001). Reduced alloreactive T‐cell activation after alcohol intake is due to impaired monocyte accessory cell function and correlates with elevated IL‐10, IL‐13, and decreased IFNγ levels. Alcohol Clin Exp Res.

[63] Shellito JE, Zheng MQ, Ye P, Ruan S, Shean MK, Kolls J (2001). Effect of alcohol consumption on host release of interleukin‐17 during pulmonary infection with Klebsiella pneumoniae. Alcohol Clin Exp Res.

[64] Ye P, Garvey PB, Zhang P (2001). Interleukin-17 and lung host defense against Klebsiella pneumoniae infection. Am J Respir Cell Mol Biol.

[65] Spinozzi F, Cimignoli E, Gerli R, Agea E, Bertotto A, Rondoni F, Grignani F (1992). IgG subclass deficiency and sinopulmonary bacterial infections in patients with alcoholic liver disease. Arch Intern Med.

[66] Jerrells TR, Smith W, Eckardt MJ (1990). Murine model of ethanol-induced immunosuppression. Alcohol Clin Exp Res.

[67] Meyerholz DK, Edsen-Moore M, McGill J, Coleman RA, Cook RT, Legge KL (2008). Chronic alcohol consumption increases the severity of murine influenza virus infections. J Immunol.

[68] Gangadaran P, Madhyastha H, Madhyastha R (2022). The emerging role of exosomes in innate immunity, diagnosis and therapy. Front Immunol.

[69] Rajendran RL, Gangadaran P, Kwack MH (2022). Application of extracellular vesicles from mesenchymal stem cells promotes hair growth by regulating human dermal cells and follicles. World J Stem Cells.

[70] Laterza OF, Scott MG, Garrett-Engele PW, Korenblat KM, Lockwood CM (2013). Circulating miR-122 as a potential biomarker of liver disease. Biomark Med.

[71] Su TH, Liu CH, Liu CJ (2013). Serum microRNA-122 level correlates with virologic responses to pegylated interferon therapy in chronic hepatitis C. Proc Natl Acad Sci U S A.

[72] van der Meer AJ, Farid WR, Sonneveld MJ (2013). Sensitive detection of hepatocellular injury in chronic hepatitis C patients with circulating hepatocyte-derived microRNA-122. J Viral Hepat.

[73] Momen-Heravi F, Bala S, Kodys K, Szabo G (2015). Exosomes derived from alcohol-treated hepatocytes horizontally transfer liver specific miRNA-122 and sensitize monocytes to LPS. Sci Rep.

[74] Saha B, Momen-Heravi F, Kodys K, Szabo G (2016). MicroRNA cargo of extracellular vesicles from alcohol-exposed monocytes signals naive monocytes to differentiate into M2 macrophages. J Biol Chem.

[75] Saha B, Momen-Heravi F, Furi I (2018). Extracellular vesicles from mice with alcoholic liver disease carry a distinct protein cargo and induce macrophage activation through heat shock protein 90. Hepatology.

[76] Momen-Heravi F, Saha B, Kodys K, Catalano D, Satishchandran A, Szabo G (2015). Increased number of circulating exosomes and their microRNA cargos are potential novel biomarkers in alcoholic hepatitis. J Transl Med.

